# A single amino acid mutation affects elicitor and expansins-like activities of cerato-platanin, a non-catalytic fungal protein

**DOI:** 10.1371/journal.pone.0178337

**Published:** 2017-05-25

**Authors:** Simone Luti, Federica Martellini, Francesco Bemporad, Lorenzo Mazzoli, Paolo Paoli, Luigia Pazzagli

**Affiliations:** Department of Biomedical, Experimental and Clinical Sciences, University of Florence, Florence, Italy; New England Biolabs Inc, UNITED STATES

## Abstract

Cerato-platanin (CP) is a non-catalytic, cysteine-rich protein, the first member of the cerato-platanin family. It is a single-domain protein with a double Ψ/β barrel domain resembling the D1 domain of plant and bacterial expansins. Similarly to expansins, CP shows a cell wall-loosening activity on cellulose and can be defined as an expanisin-like protein, in spite of the missing D2 domain, normally present in plant expansins. The weakening activity shown on cellulose may facilitate the CP-host interaction, corroborating the role of CP in eliciting plant defence response. Indeed, CP is an elicitor of primary defences acting as a Pathogen-Associated Molecular Patterns (PAMP). So far, structure-function relationship study has been mainly performed on the bacterial BsEXLX1 expansin, probably due to difficulties in expressing plant expansins in heterologous systems. Here, we report a subcloning and purification method of CP in the engineered *E*. *coli* SHuffle cells, which proved to be suitable to obtain the properly folded and biologically active protein. The method also enabled the production of the mutant D77A, rationally designed to be inactive. The wild-type and the mutated CP were characterized for cellulose weakening activity and for PAMP activity (i.e. induction of Reactive Oxygen Species synthesis and phytoalexins production). Our analysis reveals that the carboxyl group of D77 is crucial for expansin-like and PAMP activities, thus permitting to establish a correlation between the ability to weaken cellulose and the capacity to induce defence responses in plants. Our results enable the structural and functional characterization of a mono-domain eukaryotic expansin and identify the essential role of a specific aspartic residue in cellulose weakening.

## Introduction

Cerato-platanin (CP) is the first member of a recently described fungal protein family (the cerato-platanin family, CPF PF07249) consisting in non-catalytic, cysteine rich, secreted proteins that act as virulence factors, elicitors of defence responses and inducers of systemic resistance [1,2]. Until now, more than 130 sequences have been identified in filamentous fungi based on the 1–119 sequence of the mature CP. The 3D structures of CP, MpCP and Sm1 (the only structures solved until now) show a high level of homology and consist of a single domain containing the double-ψ/β-barrel fold, remarkably similar to the topology found in plant and bacterial expansins [3,4]. Plant cells secrete expansins during growth and these proteins are able to loosen and disrupt the hydrogen-bonding networks of cell wall polysaccharides without hydrolyzing them [5]. Plant expansins are small proteins of about 26 kDa consisting of two domains: the N-terminal domain (D1) that features the double ψ-β-barrel fold and is distantly related to the catalytic domain of glycoside hydrolase family-45 (GH45) and the C-terminal domain (D2), which forms an Ig-like β-sandwich fold [6]. These proteins form a long superficial groove thanks to a set of highly conserved polar and aromatic residues, able to bind the polysaccharides of the plant cell wall [7]. Expansin-like proteins have been also reported in fungi and bacteria; these proteins share the same ability to fragment cellulose aggregates with no evidence of lytic activity [8, 9]. In particular, several expansins are found in plant-pathogenic fungi and bacteria and a role in the pathogenesis process has been suggested for expansins from *X*. *campestris*, *C*. *michiganensis*, *R*. *solanacearum* and *A*. *niger* [10] and for the PcExl1 from *P*. *carotovorum* [11]. Bacterial expansins can contain extra domains such as carbohydrate binding domains, or, on the contrary, they can be single-domain fungal proteins related to the N-terminal domain of plant expansin such as the CPs and loosenins [11]. They lack the C-terminal binding domain (D2) and, consequently, have been classified as “expansins-like proteins”, to underline their ability to loosen cellulose fibrils in spite of a distantly related phylogenetic origin [7, 12, 13].

In particular, CP is an elicitor of the primary defence response and it has been classified as Microbe/Pathogen-Associated Molecular Pattern (MAMP/PAMP) that initiates a signalling cascade to prevent penetration and to restrict the growth of pathogens [14]. In fact, CP is able to induce mitogen-activated protein kinase (MAPK) cascades and the over-expression of defence genes. Furthermore the protein triggers accumulation of starch, nitric oxide (NO) and reactive oxygen species (ROS) and it activates salicylic acid and ethylene signalling pathways [14, 15]. As a PAMP, CP also induces down-expression of enzymes related to primary metabolism, reduces the photosynthesis rate and, conversely, it enhances expression of enzymes involved in GSH metabolism, in redox homeostasis and in the glucosinolate-myrosinase system, which are the premise for synthesis of defence compounds, such as camalexin [16]. A role as plant defense elicitors has also been demonstrated for other members of CPF such as BcSpl1 from *Botrytis cinerea* and Sm1 and Sm2 *Trichoderma virens* [1, 17, 18]. Despite these reports, detailed information is still missing about the mechanism by which CPF proteins interact with the plant cell wall: no pattern recognition receptor has been identified yet and only some pieces of evidence have been reported supporting the interaction between CP and the plant cuticle [19].

CPF proteins play also an important role in the physiology of fungi: CP, MpCP2 and MpCP3 from *M*. *perniciosa* and Epl1 from the biocontrol fungus *T*. *atroviride*, are expressed during rapid hyphal proliferation and spore formation and maturation [3, 18, 20]. The role of CPF proteins in the morphogenesis and the remodelling of cell wall structure, is also supported by the ability to bind insoluble chitin and soluble N-acetylglucosamine (NAG) oligomers [12, 17, 21].

Until now, studies on the bacterial EXLX1 represented the only source of information about expansin activity, thanks to the possibility to express this protein in *E*. *coli* with high yield [9]. On the contrary, expression and purification of eukaryotic expansins in prokaryotic systems is difficult [22, 23], while it was reported that eukaryotic systems such as *Saccharomyces cerevisiae* and *Pichia pastoris* may be more suitable for the expression of eukaryotic expansins [24].

The aim of this manuscript is to obtain the correctly folded, biologically active CP in the new *E*. *Coli* SHuffle [25] strain and to set up an expression model suitable for CP-like proteins containing disulfide bridges, which are otherwise difficult to express in prokaryotic system. Furthermore, we successfully subcloned a site-specific mutant and, thanks to this strain, we expressed and purified an amount of protein sufficient to carry out a characterization of the expansin-like and eliciting activities.

## Materials and methods

### Materials

Restriction enzymes, T4-DNA ligase and *Taq* DNA polymerase and 1 Kb Plus DNA Ladder were purchased from InVitrogen (San Diego, CA, USA). The Shuffle T7 Express Competent *E*. *coli* kit was from New England BioLabs. *QuikChangeLightning Site-DirectedMutagenesis Kit* was from Agilent technologies. GenElute™ PlasmidMiniprep Kit was from Sigma-Aldrich, Saint Louis, MO, USA. GST-Sepharose was obtained from GE Healthcare; the Hi Load 2.6x 60 Superdex and the C18 5 μm (4,6x250 mm) were purchased from Phenomenex. All other materials used in this study were of analytical grade and purchased from Sigma-Aldrich, unless otherwise stated.

### Methods

#### Subcloning and expression of *cp* in *E*. *coli* SHuffleT7 cells

Standard molecular biology methods were exploited to construct the expression plasmid [25, 26]. Plasmidic DNA containing *cp* gene was extracted from *E*. *coli* BL21 cells previously transformed with the *cp*-pGEX-2T plasmid in which the *cp* gene had been inserted into the unique site *BamHI* and *EcoRI* of the pGEX-2T expression vector (GE- Healthcare), downstream and in frame with the sequence encoding glutathione S-transferase (GST) [27, 28]. One hundred nanograms of plasmid DNA were used to transform competent SHuffleT7 cells according to manufacturer instructions. Cells were plated on a Luria Broth (LB) agar plate enriched with ampicillin (100 μg/mL). Four colonies were selected and grown overnight in 5mL of LB containing ampicillin at 37°C. Plasmidic DNA was extracted and sequence of *cp* gene was confirmed according to the sample submission guide of Eurofin-MWG-operon (Germany) using the primers reported in S1 Table.

Subsequently, we transferred 1 mL from the overnight culture into 100 mL of LB and we incubated the medium at 37°C unitl the OD_600_ was 0.8. Protein expression was then induced with the addition of isopropyl-β-D-1-thiogalattopyranoside (IPTG) to a final concentration of 0.4 mM. After overnight expression at 25°C, cells were harvested, resuspended in PBS containing 0.1 mg/mL lysozyme, lysed by sonication and centrifuged. Both pellet and supernatant were analysed by a 15% sodium dodecyl sulphate polyacrylamide gel electrophoresis (SDS-PAGE) and Western blot analysis to examine the presence of recombinant protein. Western blot analysis was carried out with anti-CP rabbit polyclonal antiserum and detected with HRP-conjugated anti-rabbit IgG; (Chemicon, Temecula, CA, USA) followed by an enhanced chemiluminescence reaction (Pierce Chemical, Rockford, IL).

#### Large scale expression and purification of wtCP

A strain stock, kept at -80°C, was used to inoculate 50 mL LB (20 g/L) containing 100 μg/mL ampicillin and grown overnight at 37°C under shaking to obtain wild type CP (wtCP). We then transferred 30 mL of the liquid culture into a one litre flask, that was incubated under shaking at 37°C until the OD_600_ was 0.8. Protein expression was then induced with 0.4 mM IPTG; expression was carried out overnight at 25°C, as reported above for the small-scale expression. Cells were harvested by centrifugation and the pellet was resuspended with 40 mL PBS containing 1 mg/mL lysozyme. After 30 min of incubation at 37°C, the lysis was performed by five sonication cycles at 50 kHz. The lysate was centrifuged at 12,000 rpm for 30 minutes and the supernatant fraction, containing the fusion protein GST-CP, was recovered.

The lysate (40 mL) was applied to a 10 mL GSH-Sepharose column equilibrated with 50 mM Tris-HCl buffer at pH 8 containing 150 mM NaCl. The column was eluted by adding 5 mM glutathione and the pooled fractions were concentrated to 5 mL for thrombin treatment. Five hundred microliters aliquots were used to identify the best among a set of digestion conditions with incubation times ranging from 3h to overnight and urea concentrations ranging from 1 M to 0.0125 M. The best condition for the cleavage of the fusion protein corresponded to the overnight incubation at 37°C in the presence of 2.5 U/mL thrombin, 2 mM CaCl_2_, 12 mM urea. The cleavage products were applied onto a 100 x 3.5 cm Superdex G75 column equilibrated with 50 mM Tris-HCl buffer pH 8.0 containing 150 mM NaCl. The presence of CP during the purification steps was monitored by 15% SDS-PAGE and protein concentration was determined by the bicinchoninic acid method (BCA, Pierce Chemical, Rockford, IL).

Native CP was obtained, according to the method described by Pazzagli et al., starting from the cultural filtrate of *C*. *platani*, which was lyophilised and subjected to gel filtration and RP-HPLC chromatography [29]. This protein was used as positive control in circular dichroism (CD) measurement and biological assays.

#### Site direct mutagenesis, expression and purification of the D77A mutant

Primers for mutagenesis were designed considering an optimal length of 25–45 residues and a melting temperature above 78°C. Eighty nanograms of template *cp*-pGEX-2T were mixed with 125 ng of the primers (S1 Table) to obtain the mutation of GAC (coding for Asp) in GCC (coding for Ala), exploiting the QuikChange Lightning Site-Directed Mutagenesis Kit and XL 10-Gold ultra-competent cells. Plasmidic DNA was extracted, sequenced at Eurofins-MWG-operon and subsequently used to transform competent SHuffleT7 cells as reported above for the wild type protein. The pellet obtained after the lysis of the transformed cells was frozen at -80°C. Large scale expression and purification of the mutCP (D77A) was performed according to the same protocol used for wtCP, with some modifications. The cleavage products from thrombin digestion were purified on a C18 column (Phenomenex, 4,6 ×250 mm; 5 μm; Grace, Columbia, MD) by using a Trifluoroacetic acid/acetonitrile gradient. 15% SDS-PAGE and BCA assay were performed to check the yield of purification and purity of the obtained protein.

In a separate set of experiments, purification of CP from the inclusion bodies was performed. Briefly, the frozen pellet obtained after the lysis of the transformed cells was resuspended in 25 mL of the lysis buffer containing 3.5 μL of 10 mM PMSF, 14 μL of 10 mg/mL of lysozyme and 1% Nonidet NP-40. After 1h incubation at 4°C, 15 μL of DNA-ase (1 mg/mL) and 15 μL of 1 M MgSO_4_ were added and the mixture was incubated for 40 min at 37°C under shaking. The supernatant obtained by centrifugation was analysed by 15% SDS-PAGE and the pellet was treated with 6 M guanidine hydrochloride. The solubilized protein was refolded after a dialysis step and analysed by circular dichroism.

#### Mass spectrometry analysis

Twenty picomoles of native CP (nCP), wtCP and mutCP were dissolved in 50% v/v acetonitrile containing 0.05% v/v trifluoroacetic acid, diluted 1:1 in a saturated sinapinic acid matrix, applied on the anchor-chip target plate and allowed to dry. Samples were analyzed with a Ultraflex III MALDI-TOF mass spectrometer (Bruker Daltonics, Bremen, Germany) by using the Flex Control 3.0 data acquisition software. Spectra were obtained in LINEAR mode over the m/z range 1500–20000 for a total of 500 shots. The instrumental parameters were chosen by setting the ion source 1 at 25 kV, ion source 2 at 21.5 kV, the pulsed ion extraction at 20 ns. The instrument was externally calibrated prior to analysis using the Bruker Peptide Calibration standard kit.

#### Circular dichroism spectra and 3D models construction

***Far UV CD spectra***: nCP, wtCP, and mutCP were dissolved in water to a final concentration of 6.25 μM. CD spectra were recorded in the far-UV region from 190 to 260 nm using a JASCO J-810 spectropolarimeter (Tokyo, Japan). The spectra were acquired using a 1-mm path length quartz cell, a band-width of 1 nm, a response time of 0.5 s, a data pitch of 0.5 nm and a scanning speed of 100 nm/min. Each spectrum was the average of six consecutive scans followed by subtraction of the blank spectra. The observed signal–in mdeg- was converted to mean residue molar ellipticity (expressed in deg cm^2^ /dmol) according to the formula [θ] = CD / [(c/m) 10 d n], where CD is the observed signal, c is the protein concentration in g/L, m is the molar mass in g/mol, d is the path length in cm and n is the number of amino acid residues.

***Near UV CD spectra***: wtCP and mutated rCP were dissolved in water to a final concentration of 1.5 mg/mL. CD spectra were recorded in the near-UV region from 270 to 300 nm using the JASCO J-810 spectropolarimeter. The spectra were acquired using a 1-mm path length quartz cell, a band-width of 2 nm, a response time of 2 s, a data pitch of 0.5 nm and a scanning speed of 10 nm/min. Each spectrum was the average of 80 consecutive scans followed by subtraction of the blank spectra. Ellipticity was expressed as mean residue molar ellipticity (θ) (deg cm^2^dmol^−1^) as reported for far UV spectra.

***3D models of CP*.** Models of wt and mutCP were obtained using Pymol (The PyMOL Molecular Graphics System, Version 1.8 Schrödinger, LLC). The CP file was downloaded from pdb (2kqa.pdb). Every structure was minimized using the optimize plugin with the following parameters [30]: forcefield, GAFF, method, conjugate gradient, steps 500 and convergence 0,0001. All the 20 structures were analysed to assess the formation of H bonds. The structures were mutated with mutagenesis wizard, and they were again energy minimized.

***Equilibrium denaturation experiment*.** We tested conformational stability of wtCP and mutCP with guanidine hydrochloride (GndHCl) equilibrium denaturation experiments. Briefly, 21 samples containing the tested variant at a concentration of 0.04 mg/ml were prepared in the presence of GndHCl concentrations ranging from 0 to 5.6 M. We incubated the obtained samples for 30 minutes at 25°C. Fluorescence spectra from 300 to 500 nm were then acquired in 10 x 2 mm cells (Hellma) with a PerkinElmer LS-55 spectrofluorimeter (Waltham, MA, USA), using an excitation wavelength of 280 nm and excitation and emission slits of 5 nm. The centre of mass (COM) of each spectrum was calculated as COM = (∑_i_ *F*_i_ *λ*_i_) / (∑_i_ *F*_i_), where *F*_i_ is the fluorescence emitted at a wavelength of *λ*_i_. The obtained values were plotted Vs GndHCl concentration and analysed with the method published by Santoro & Bolen [31], to yield the free energy change following denaturation (Δ*G*_U-F_^H2O^), the concentration of middle denaturation (*C*_m_) and the dependence of free energy change on [GndHCl] (*m*).

#### Biological activity

***Phytoalexins production***. The ability to induce the synthesis of phytoalexin-like molecules from the leaves of the non-host *Arabidopsis thaliana* (Col. 0) was tested as previously described [14]. Briefly, five 10 μL drops of 150 μM nCP, wtCP and mutCP were applied on the lower surface of *A*. *Thaliana* leaves and put into a moist chamber under continuous light. Water controls were performed for each sample and, after 24 h of incubation, droplets were recovered. Fluorescence was recorded with the LS-55 spectrofluorimeter using excitation and emission wavelengths of 320 mn and 386 nm, and excitation and emission slits of 5 nm. Results were expressed as fluorescence intensity/drop. Data obtained were the average of six independent measurements. Statistical analysis was performed by unpaired *t-test* (treated vs. control).

***H***_***2***_***O***_***2***_ ***production*.** *A*. *thaliana* leaves were treated with wtCP and mutCP and H_2_O_2_ was visualized *in situ* according to Lombardi et al. [15]. Briefly, 10 μL drops of a 150 μM solution of each protein were applied on leaves and incubated for 24h in a moistly chamber at room temperature. After incubation, droplets were removed and H_2_O_2_ production was visualized by adding the specific probe 2’-7’ dichlorodihydrofluorescein diacetate (DCFH2-DA; Sigma-Aldrìch, Saint Louis, MO, USA), which is rapidly oxidized to highly fluorescent dichlorofluorescein (DCF) in the presence of H_2_O_2_. Leaves were then incubated in a 20 mM sodium phosphate buffer at pH 6.8 containing 10 μM DCFH-DA, at room temperature for 1h. After staining, the samples were washed twice in fresh buffer to remove the excess of fluorophore, and mounted in buffer on microscopic slides. Green fluorescence was then observed, using an excitation wavelength of 488 nm under a confocal Leica TCS SP5 scanning microscope (Leica, Mannheim, Germany).

***Activity on cellulose*.** The ability of wtCP and mutCP to weaken filter paper was tested on Whatman no. 1 filter paper (GE Healthcare) as previously reported [12] with some modifications. Filter paper was cut into 0.6 cm diameter discs of about 4 mg each, and a single disc was incubated in 0.5 mL of 50 mM sodium acetate buffer (pH 5.0) containing the proteins at a concentration of 30 μM. Buffer only or buffer containing the protein bovine serum albumin (BSA) at the same concentration were used as negative controls. The experiments were performed in 2 mL tubes sealed with laboratory film and incubated at 38°C, for 48h, onto an HLC thermomixer at 700 rpm. At the end of incubation, pictures were taken, discs were removed and the absorbance at 500 nm was measured. Each measurement was taken immediately after shaking the suspension in the measuring cuvette, and three different measurements were acquired for every suspension.

## Results

### Cloning and expression of *cp* gene in *E*. *coli* SHuffleT7 cells

Four colonies of *E*. *coli* Shuffle T7 transformed with the *cp*-pGEX-2T plasmid were grown on liquid media and subjected to plasmid extractions (S1A Fig). Expression of the recombinant protein in *E*. *coli* SHuffleT7 was analysed by SDS-PAGE. GST-CP content in the supernatant and pellet fractions was assessed by anti-CP antibodies. The fusion protein was obtained in both soluble and insoluble fraction (S1B Fig). Colony 1 was chosen for the highest yield in the supernatant fraction and used for large scale expression; colony 3 was discarded since a large amount of protein had been included into the pellet.

### Site direct mutagenesis

Asp77 is a key residue in CP and is conserved throughout the members of CPF (S4 Fig). Therefore, to investigate the role played by the carboxyl group of this residue, a CP protein variant, carrying the mutation D77A (mutCP) was produced. MutCP was obtained by site direct mutagenesis of the GAC codon (coding for Asp77) in GCC (coding for Ala) by the use of XL10 Gold *E*. *coli* competent cells. Transformation and mutation were assessed by agarose gel electrophoresis and sequencing, respectively. The plasmid containing mutCP was used to transform competent SHuffleT7 cells. Expression of recombinant protein was monitored in the pellet and in supernatant fractions as reported for the wtCP and the colony that exhibited the highest expression level of soluble CP was chosen for large scale production (S1C and S1D Fig).

### Large scale expression and purification of wtCP and mutCP

**Fig 1:**
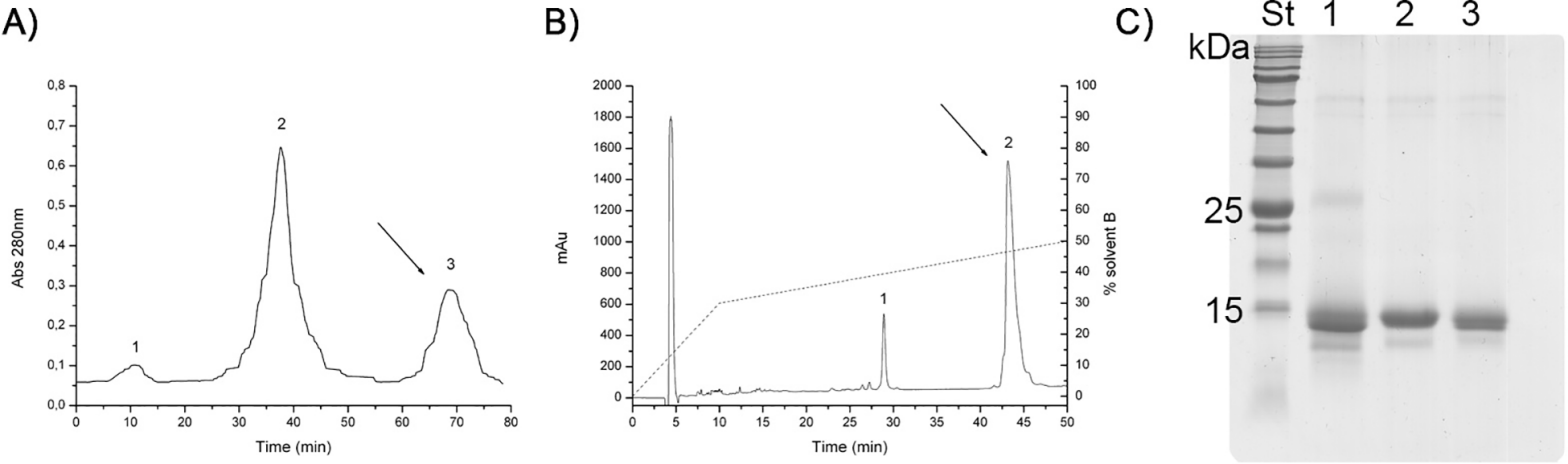
Purification of wt and mutCP. A) Gel filtration chromatography. Purification of wtCP on 100 x 3.5 cm Superdex G75 column; 50 mM Tris-HCl buffer pH 8.0 containing 150 mM NaCl was used. Peak 1: GST-wtCP; Peak 2: GST, Peak 3: wtCP. B) RP-HPLC purification of mutCP. The column was Phenomenex, 4,6 ×250 mm; 5 μm;Solvent A, 10 mM TFA in water; Solvent B, 10 mM TFA in acetonitrile. Flow rate was 0.8 ml/min. The elution was performed by an acetonitrile gradient as indicated by the dotted line (……). Peak 1: mutCP; Peak 2: GST-mutCP. C) 15% SDS-PAGE of the pure proteins. Lane St: Prestained Protein SHARP*MASS* VI marker (Euroclone); Lane 1: native CP; Lane 2: recombinant wtCP; Lane 3: D77A mutCP. 2μg of each protein were applied; gel was stained with Comassie Brilliant Blue.

The fusion protein GST-wtCP was recovered after cells lysis and purified with a GSH-Sepharose resin. Removing the tag was performed by thrombin digestion in the presence of urea to unmask the cutting site probably hidden in the fusion protein. In fact, thrombin alone is not able to cut the fusion protein, and urea is needed, even if in low concentration, to obtain an high digestion yield (S2 Fig). The final purification step consisted of gel filtration by Superdex column and allowed us to obtain the pure protein (Fig 1A).

Expression and purification of the mutCP (D77A) was carried out with a slightly modified version of the protocol applied to purify wtCP: rather than being subjected to gel filtration, the cleaved product obtained after thrombin digestion was applied to a Reverse Phase HPLC column (Fig 1B). This was necessary because the purification yield of mutCP was lower than the yield of wtCP, probably due to the tendency of the mutated protein to precipitate into inclusion bodies, as highlighted by SDS-PAGE, (S3 Fig). Although different protocols were employed and tested for their ability to recover the protein from the inclusion bodies, only a little increase in the purification yield was obtained. Furthermore, mutCP recovered from inclusion bodies was not correctly folded, and it was not further investigated.

Purity of the wtCP and mutCP was analysed by SDS-PAGE: a single band corresponding to the molecular weight of about 12 kDa was observed for the two proteins (Fig 1C). A comparison among the yields of the different purification procedures is reported in Table 1.

**Table 1:** Purification yield of wild type and mutated CP starting from 1L of induced culture. Values are the means of data from four independent experiments ± s.d.

Purification step	wtCP (mg)	mutCP (mg)
**E.coli cultured cells**	183,4 ± 18,7	169,2 ± 9,8
**Affinity Sepharose-GSH**	20,7 ± 3,6	3,0 ± 0,8
**Gel filtration Superdex G75**	8,9 ± 1,5	---
**Reverse Phase HPLC**	----	0,42 ± 0,1

### Structural characterization of wtCP and mutCP

**Fig 2:**
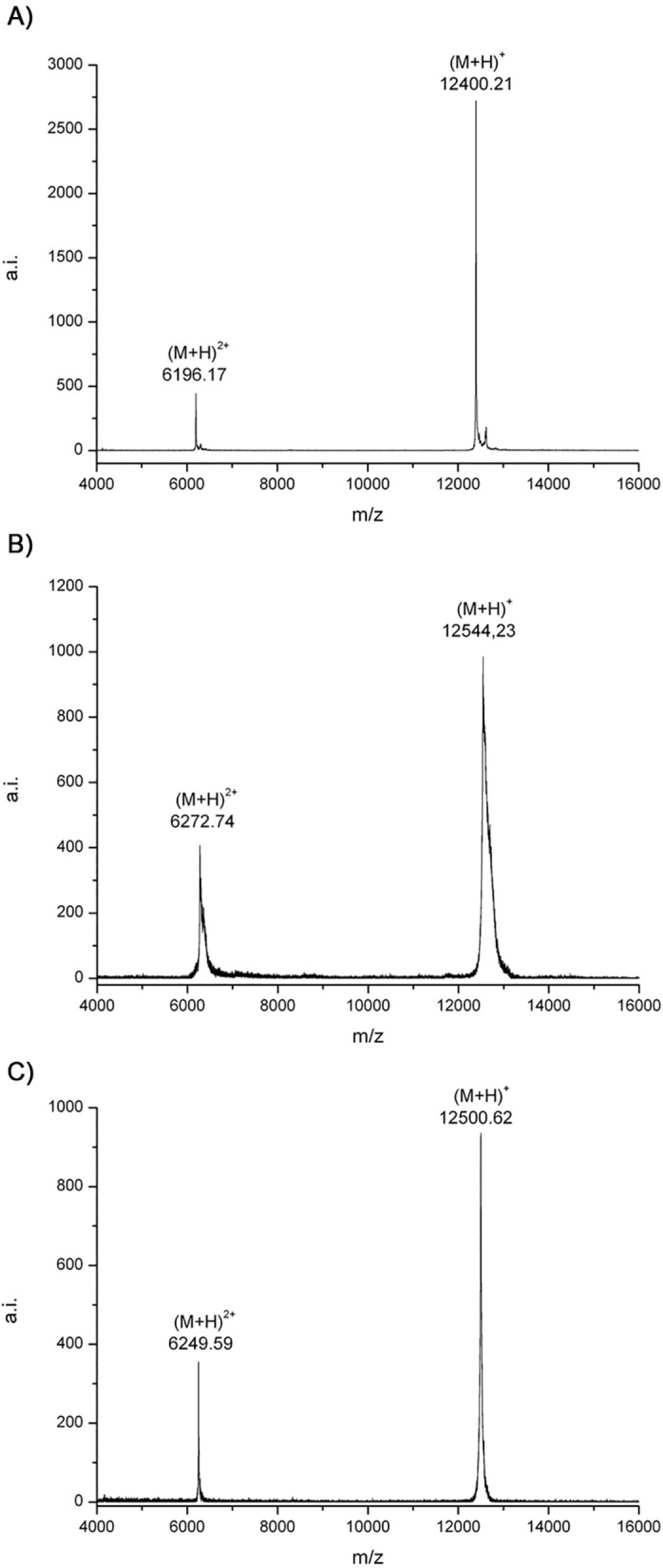
MALDI–TOF mass spectrometry. Twenty picomoles of proteins were dissolved in 50% acetonitrile containing 0.05% TFA, diluted 1:1 in saturated sinapinic acid matrix and analysed on a MALDI–TOF mass spectrometer. A) nCP. B) wtCP. C) mutCP.

The exact molecular weight of wtCP and mutCP was determined by means of mass spectrometry and the values were compared with that of the native form. As shown in Fig 2 the molecular mass of nCP and wtCP, were 12399 Da and 12543 Da (Δm = 144 Da), respectively, and this result was in agreement with the presence of two extra residues, Gly-Ser, at the N-terminus of wtCP. The mass of mutCP is 12500 Da and this result is in agreement with the difference between Asp and Ala (Δm = 44Da) (Fig 2). In conclusion, results of mass spectrometry confirm that the masses of the recombinant proteins purified correspond to the expected values, i.e. the values calculated from primary sequences.

**Fig 3:**
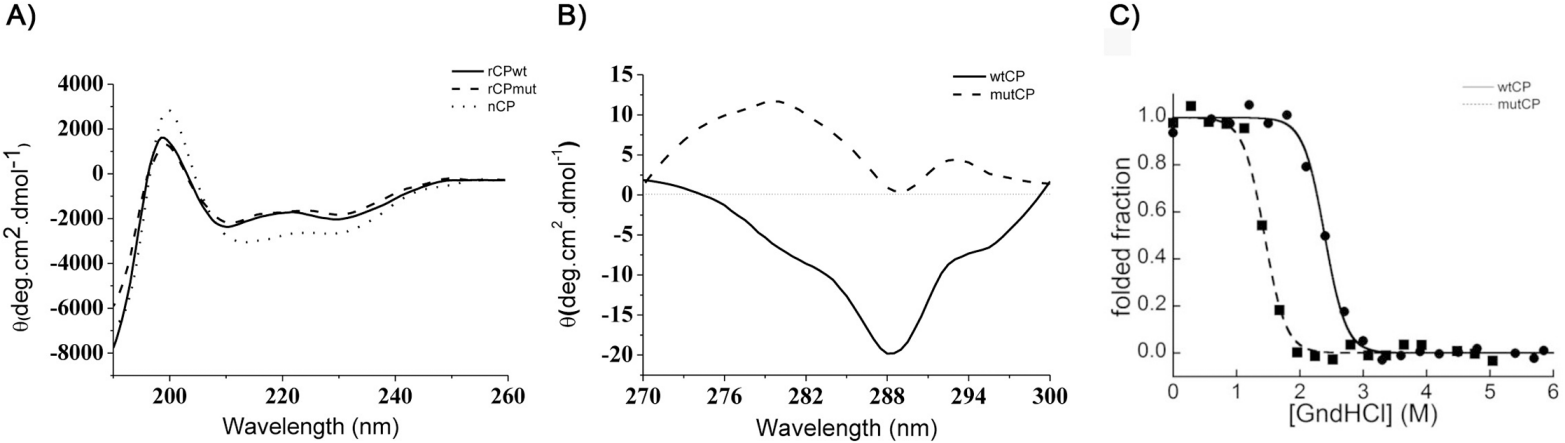
Structural characterisation of CP. A) Far-UV CD spectra of 6.25 μM nCP, wtCP and mutCP. B) Near-UV CD spectra of 1.5 mg/mL wtCP and mutCP. All samples were dissolved in 10 mM Na-phosphate buffer.C) equilibrium denaturation experiment of 0.04 mg/ml wtCP and mutCP in the presence of GndHCl concentrations ranging from 0 to 5.6 M.

The secondary structure of the purified proteins was analysed by far-UV CD spectra. Fig 3A shows that far-UV CD spectra of wtCP and mutCP are overlapped, thus suggesting that these two variants share the same secondary structure. Near-UV CD spectra of wtCP and mutCP were also acquired to evaluate the impact of the mutation on the local environment surrounding aromatic side chains of the protein. As shown in Fig 3B, the spectra of the two proteins were different especially between 275 and 282 nm, where tyrosine absorbs.

**Fig 4:**
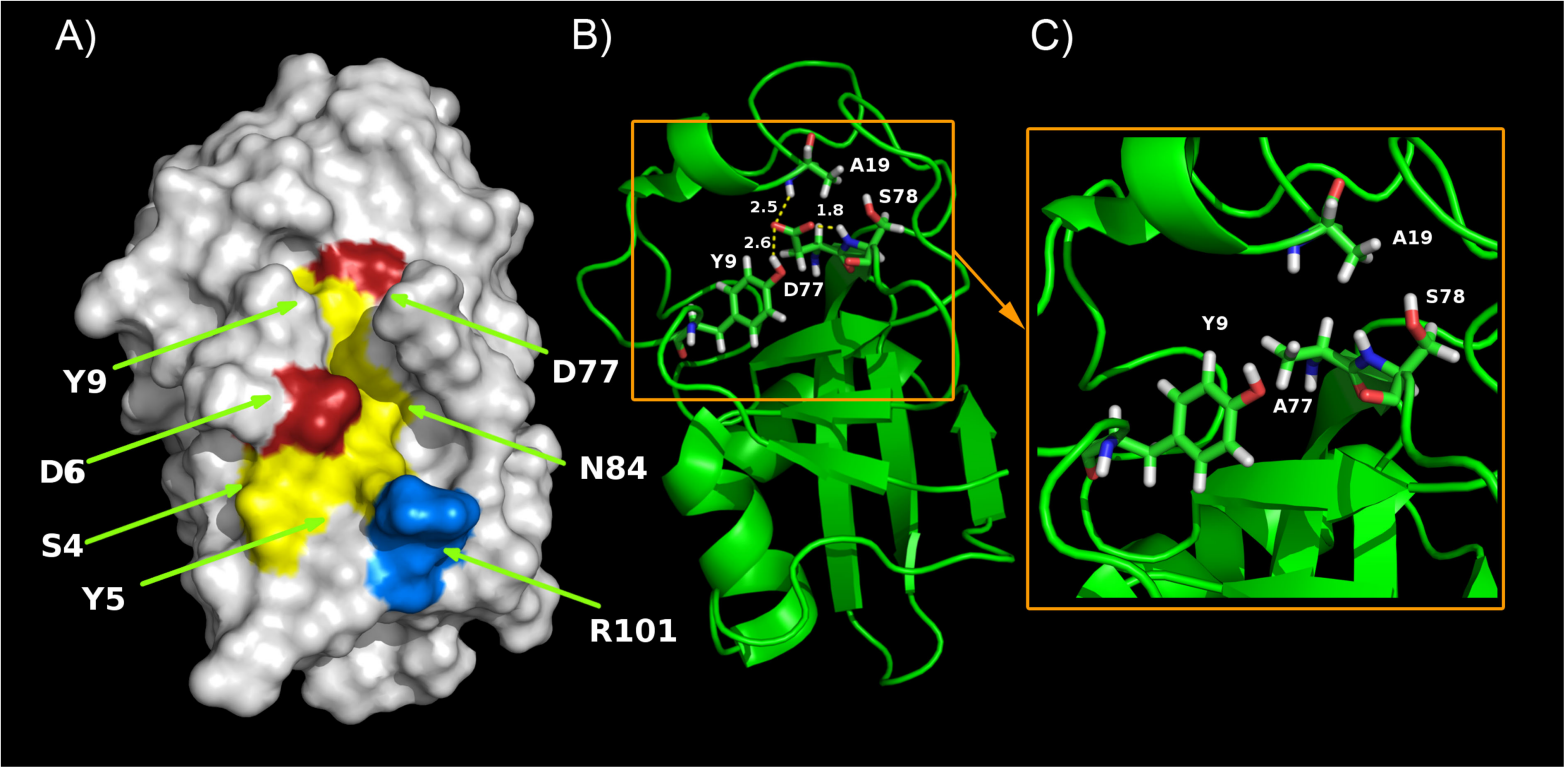
3D model of CP structure. A) Surface representation. Coloured residues are located in the putative oligosaccharide binding region. Colours shown conserved amino acid along all CPF members. Red for acid residues, blue for basic residues and yellow for-polar residues. B) H bonds distances in wtCP. The bonds between D77-Y9, D77-S78 and D77-A19 are indicated. Yellow rectangle indicates the zoomed region in C. C) H bonds in mut CP. Lack of H bonds in mutCP with A77 instead D77.

In order to further evaluate the overall fold of the mutated protein, we gauged conformational stabilities of wtCP and mutCP by means of equilibrium denaturation curves. The results of these experiments, shown in Fig 3C, reveal that wtCP is a highly stable protein. While the protein resists denaturation at GndHCl concentrations ranging from 0 to 2 M, a single cooperative unfolding transition can be observed between 2 and 4 M GndHCl. Analysis of the experimental curve with the method by Santoro & Bolen [31] provides thermodynamic parameters associated to this transition. The conformational stability (ΔG_U-F_^H2O^) of wtCP is 35.2 ± 0.4 KJ mol^-1^, with a concentration of middle denaturation (*C*_m_) of 2.4 ± 0.2 M and a *m* value of 14.7 ± 0.3 KJ mol^-1^ M^-1^. In contrast, removal of the carboxyl group of Asp 77 results in a strongly destabilised variant. mutCP exhibits a cooperative transition, which is however complete in the presence of 2 M GndHCl. ΔG_U-F_^H2O^ obtained for mutCP is 21.2 ± 0.4 KJ mol^-1^, with a *C*_m_ of 1.4 ± 0.2 M and a *m* value of 14.7 ± 0.3 KJ mol^-1^ M^-1^ (Fig 3C). While these results confirm that mutCP is globally folded in the absence of denaturant, they suggest the D77A mutation caused a decrease in protein stability. In an effort to identify the regions affected by the mutation introduced with mutCP, we obtained 3D models of wtCP and mutCP with PyMol. Fig 4A shows the D77 located in a polar pocket defined by the S4, Y5, D6, Y9, N84 and R101 residues which are highly conserved among all the CPF members (S4 Fig). The low energy structures obtained from NMR data reveal the presence of H bonds between D77-Y9 and D77-S78 of wtCP. A further H bond can be observed between the Asp chain and backbone NH group of A19 as shown in Fig 4B, where the residues involved in H bonds formation and bond distances are shown. The effect of the D77A mutation on hydrogen bonds formation is shown in Fig 4C: no such bonds can be detected in the mutated protein.

### Biological activities of wtCP and mutCP

**Fig 5:**
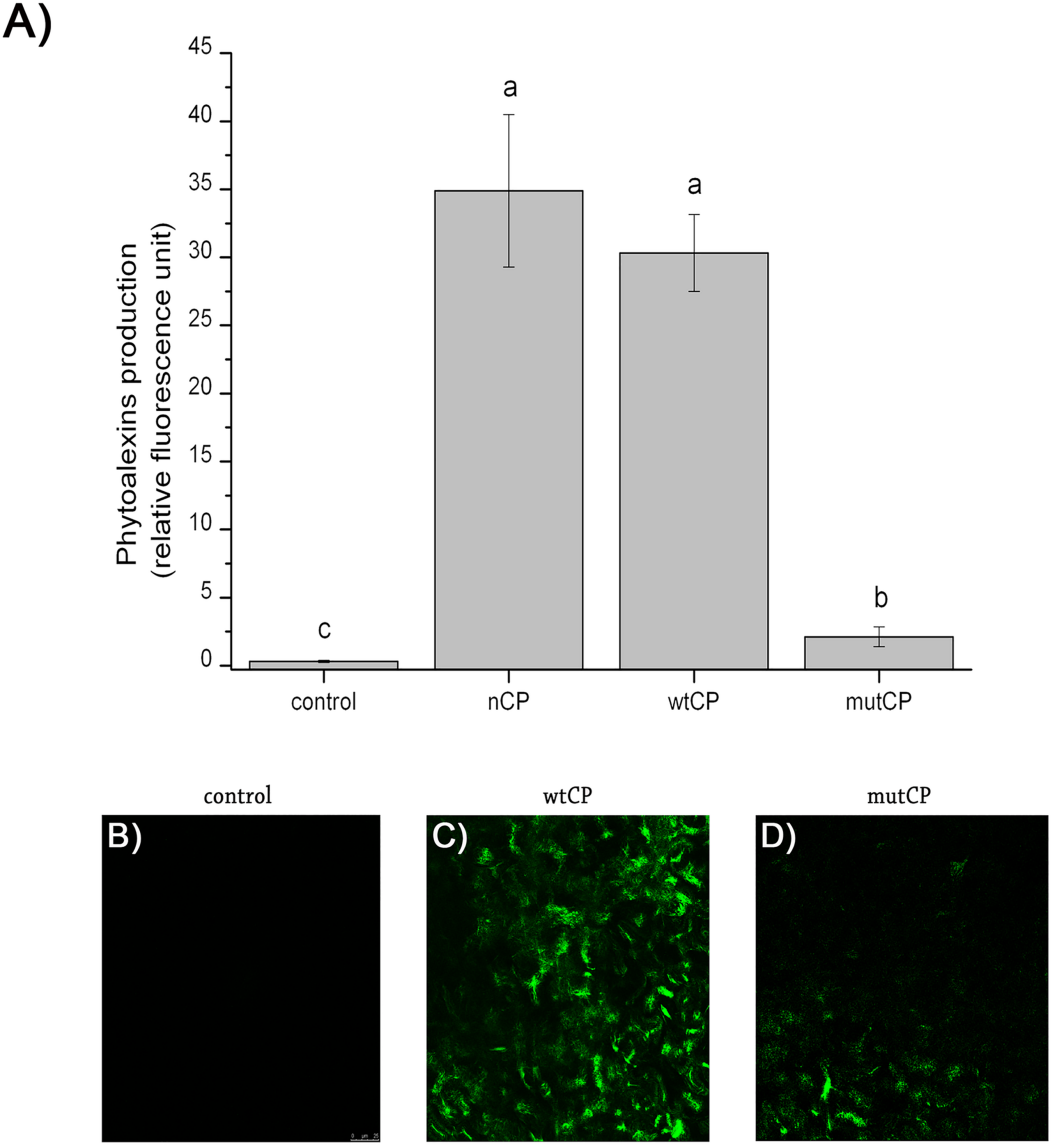
Eliciting activities of CP on *Arabidopsis* leaves. A) Phytoalexins production. Fluorescence emitted by 150 μM of nCP, wtCP and mutCP was assayed after 24h of incubation. Data are the mean ± s.d. of values obtained by six independent experiments performed in duplicate. Values marked with different letters are significantly different at p< 0.01 according to the *t-test*. B) ROS production. *A*. *thaliana* leaves treated for 24h with H_2_O (or with150 μM wtCP (C) and mutCP (D). H_2_O_2_ was visualized in situ by the fluorescent probe DCFH2-DA.

In a first set of experiments, we compared the ability of native CP and wtCP to induce the synthesis of phytoalexins-like molecules in *A*. *thaliana* leaves. While native CP was used as positive control, production of phytoalexins induced by wtCP is not significantly different from the production observed in the presence of native CP, thus confirming the ability of the *E*. *coli* SHuffle recombinant system to express correctly folded disulphide-bridges rich proteins (Fig 5A). In contrast, mutCP induces a very low synthesis of phytoalexins, with values comparable to the negative control (Fig 5A).

Next, we assayed the ability of wtCP and mutCP to induce production of H_2_O_2_. We exploited fluorescence microscopy to detect ROS synthesis on *Arabidopsis* leaves. Fig 5C shows the results obtained for wtCP: the vast majority of the visualized field shows strong signals in green fluorescence. On the contrary, leaves treated with mutCP show a low emitted green fluorescence that can be recovered only in a restricted area, while a vast proportion of the analysed surface shows no signals, as observed with the control (Fig 5D and 5A respectively).

**Fig 6:**
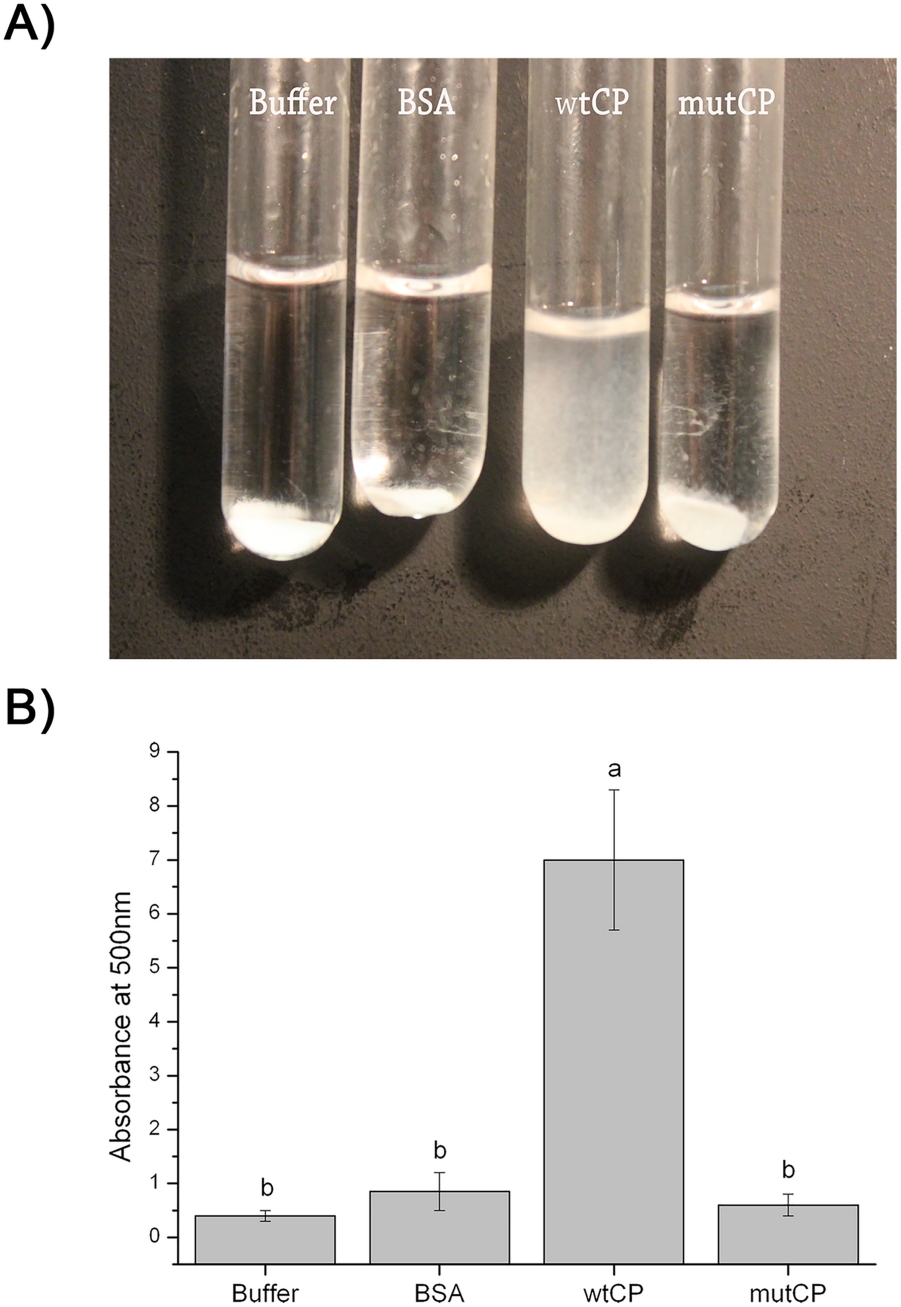
Weakening activity of CP on filter paper. A) Releasing of paper fragments from the paper disc Each tube contains a filter paper disc in 0.5 mL of 50 mM sodium acetate buffer and 30 μM of wtCP or mutCP. Buffer only or buffer containing 30μM BSA at the same concentration were used as negative controls. Weakening activity was visible as paper fragments released in suspension from the paper disc after 48h, at 38°C, with shaking at 700 rpm. B) Quantification of the paper fragments produced. Absorbance at 500 nm was measured on a Ultraspec 2000 (Pharmacia biotech) spectrophotometer. Error bars indicate the standard deviation of measurements from three separated experiments. Values marked with different letters are significantly different at p< 0.05 according to the *t-test*.

Last, we compared wtCP and mutCP for another well know capability of cerato-platanin, i.e. the weakening activity on cellulosic materials, which is a hallmark of the expansin-like activity. wtCP produced in *E*. *coli* SHuffle was able to weaken filter paper, as revealed by the turbidity of the solution (Fig 6A). The addition of mutCP to the filter paper did not cause any visible paper fragmentation, suggesting the loss of expansin-like activity. We then quantified the activities of wtCP and mutCP by evaluating absorption at 500nm: absorption measured in the presence of wtCP is comparable to the values obtained for the native protein [12]; conversely, the activity of mutCP appears to be similar to the negative control (Fig 6B).

## Discussion

Interest in CP has been recently raising partially because CPF is continuously increasing in new members that share many conserved residues, even when members of distantly related origin are compared [2]. Moreover, although CP lacks hydrolytic activity, the double-ψ/β-barrel domain is structurally related both to the catalytic domain of glycoside hydrolase family-45 and to the D1 domain of plant and bacterial expansins [4, 12]. In this regard, CP and other proteins, such as swollenins and loosenins, produced by fungi, have been recently defined “expansin like proteins” because of their sequence relatedness with expansins and their ability to weaken cellulose aggregates with no evidence of lytic activity [10,13, 32]. In particular, CP lacks the D2 domain and, therefore, can be defined a mono-domain expansin. The ease of producing the protein in high yield in *P*. *pastoris* as well as the extraordinary heat stability, make this protein suitable for future potential applications in increasing cellulose loosening rate for example during biofuel production [12].

In order to undertake a structure/function relationships study, here we rationally designed the mutant of a key residue in CP structure to test its role in cellulose weakening. To achieve this goal, the expression of CP in the yeast *Pichia pastoris* has to be changed because of difficulties in carrying out experiments of site-direct mutagenesis in eukaryotic systems. Canonical prokaryotic systems (such as *E*. *coli* BL21) had not allowed correctly folded CP to be obtained because of improper formation of disulfide bridges: only after one further refolding step CP reaches its correct fold, but poor yield and length of a cumbersome purification procedure make the method unsuitable [28]. In 2012 Lobstein and colleagues have developed a new strain of *E*. *coli* called Shuffle, able to express correctly folded disulfide bonded proteins within its cytoplasm. This remarkable result was achieved thanks to diminished reductive pathways and to the insertion of signal sequence-less disulphide bond isomerase [25]. Recently, many papers reported the successful use of *E*. *coli* SHuffle for expression of disulphide bridged proteins that can be purified in high yield [33, 34, 35]. Consequently, we chose the Shuffle T7 strain to express CP and to produce the mutated protein. The recombinant CP obtained (wtCP) was correctly folded and biologically active. Until now, the heterologous expression of eukaryotic expansins and expansin-like proteins proved to be very difficult [24]; for this reason, we think that the method presented here is a good starting point to obtain other recombinant fungal and plant expansins.

Starting from the observation that one specific Asp residue (D77) is conserved in expansins and expansin-like proteins and that this residue seems to play a major role in the loosening activity [9, 24, 36], we decided to mutate the D77 residue, for several reasons: i) D77 is conserved in all the members of CPF identified so far (S4 Fig); ii) D77 may have a role similar to that played by D82 of the BsEXLX1expansin D1 domain and by the catalytic proton donor (D121) of GH45-endoglucanases [9], iii) D77 interacts with Tyr9, another highly conserved residue throughout CPF, and it is positioned in a flat and superficial groove on one side of the β-barrel, which presents several polar and aromatic residues suitable for sugar carbohydrate binding.

The purification yield of mutCP is lower because of the incorporation of the protein into inclusion bodies, which is probably a result of the destabilizing effect induced by the mutation. The D77A mutation shifts the hydropathy value (GRAVY) from 0.002, calculated for wtCP, to 0.046. Thus, the mutation affects the solubility of the protein due to the loss of negative surface density.

However, the amount of purified mutCP was sufficient to enable structural and functional characterization to be carried out in comparison with the wtCP. As far as the structure of mutCP is concerned, the far UV spectra indicate that the mutation leaves secondary structure unaffected. On the contrary, the near UV spectra suggest a difference in the packing of aromatic residues and, consequently, in the tertiary structures, in agreement with the idea that the near UV CD spectrum of a protein provides a fingerprint of the tertiary structure [37]. The difference observed in the band located between 275 and 282 nm is assigned to the Tyr signal: the loss of the hydroxyl group of D77 abolishes an H bond between D77 and Y9, thus modifying the Tyr peak. Pieces of evidence on the H bond formation between the two highly conserved residues D77-Y9 have been reported with NMR by de Oliveira et al. [4] and are of outstanding importance since this H bond may be structurally related to the D82-T14 bond of the D1 domain of the bacterial expansin BsEXLX1 [7, 9]. The importance of D77 is also confirmed by the putative involvement of the carboxyl group in another H bond with NH group of A19. In conclusion, our data indicate that the carboxyl group of D77 plays a pivotal role in the formation of H bonds that stabilize the structure, being located in the hind region between the ψ/β barrel and the random coil region. Therefore, the D77A mutation explains the differences observed in near CD-spectra, as also suggested by the equilibrium denaturation experiments: mutCP, albeit globally folded in absence of denaturant, shows a decrease in conformational stability which is in agreement with the partial misfolding of the hinge region, probably due to the disruption of the H-bond discussed above.

To start a structural/functional relationship study, we monitored the expansin-like activity and the elicitor activity in inducing defence responses during plant-protein interaction of the D77A mutant. Since CP has been characterized as PAMP, we firstly assessed the ability to induce synthesis of antimicrobial secondary metabolites such as phytoalexins and H_2_O_2_ from the lower surface of *Arabidopsis* leaves. In fact, ROS production and phytoalexin synthesis are among the best characterized defence responses induced by PAMPs [38, 39, 40]. In both cases, the mutCP does not produce any detectable PAMP activity: amount of phytoalexins synthesized by mutCP is comparable to the control upon plant interaction; alike, the production of H_2_O_2_ is extremely reduced when leaves are treated with mutCP. This observation suggests a major role played by the mutated residue in CP activity and indicates that the structural changes induced by the D77A mutation strongly affect the ability of CP to induce defence responses. This is in agreement with previous findings indicating an interaction of D77 with N-acetylglucosamine oligomers used to mimic chitin in NMR binding experiments and, more in general, with the role of elicitors in oligosaccharide interaction [4, 41, 42].

Using filter paper diskettes, we observed that wtCP is able to form a suspension of released particles, as observed before for CP from *P*. *pastoris* [12]. Conversely, mutCP does not exhibit any loosening activity. This finding is in agreement with the observation that an aspartic residue is conserved in expansin and expansin-like proteins and with the complete loss of weakening activity in the D82A mutant from EXLX1 expansin [36, 43, 44].

Simulation studies suggest that BsEXLX1 may be able to migrate on cellulose and disrupt hydrogen bonds by twisting glucan chains. BsEXLX1 can slide on the hydrophobic surface of crystalline cellulose by interacting with the flat aromatic surface located in the domain D2; the D82 residue belonging to D1 domain would instead be responsible for hydrogen bond formation with a free glucan chain in twisted conformation [45]. Although CP, which lacks the D2 domain, is not able to bind cellulose [12], the protein is able to fragment filter paper, and this activity is lost in the D77A mutant. This finding, together with the observation that the pH optimum for the activity of CP on cellulose is 5, a pH value at which D77 is likely to be protonated, lends support to the hypothesis of a primary role of D77 in hydrogen bond formation and justifies the definition of CP as a novel mono-domain expansin. However, a role for the alteration of the structure in the hinge region, as evidenced by CD spectra and equilibrium denaturation experiments, cannot be ruled out presently: the loss of hydrogen bonds and the increased hydrophobicity in the active area–in which all key residues are located- can contribute to impair the ability of CP to weaken cellulose fibrils. Therefore, even if mutCP is globally folded, the D77A mutation induces an alteration in conformational stability that may underlie, or at least contribute to the inactivation induced by mutation.

In conclusion, our results highlight the role of D77 both in PAMP and in expansin-like activities, enabling us to establish a correlation between the ability to degrade cellulose and the ability to induce defence responses in plants. In other words, a loosening of the host cell wall could be a pre-requisite for host-pathogen interaction and, based on conservation of key residues in CPF, this observation may be valid not only for CP but also for the other CPF proteins. The lack of an identified receptor for CP makes difficult to understand, at a molecular level, the inability of mutCP to activate defences. However, our results let us to hypothesize that the correct fold of the hinge region between the random coils and the β-barrel core, as well as the hydroxyl group of D77 in the shallow groove, are important for biological activity of CP.

## Supporting information

S1 Table Sequence of primers used in molecular cloning and mutation of the *cp* gene. (DOCX)

S1 Fig Cloning and expression of *cp gene* in *E. Coli* SHuflle cells. A-B) wtCP: A) 1% Agarose gel electrophoresis. Line: (St)1 Kb Plus DNA Ladder; (1–4) colony number. B) Western blot analysis probed with anti-CP of *E*. *coli* SHuffle lysate of 1–4 colonies; (a) insoluble fraction, (b) soluble fraction.C-D) mutCP: C) 1% Agarose gel electrophoresis. Line: (St)1 Kb Plus DNA Ladder; (1–4) colony number. D) Western blot analysis probed with anti-CP of *E*. *coli* SHuffle lysate of 1–4 colonies; (a) soluble fraction, (b) insoluble fraction.
(TIF)

S2 Fig 15% SDS-PAGE of thrombin digested GST-CP protein. Lane 1: GST-CP+0M Urea; Lane 2: GST-CP+1M Urea; Lane 3: GST-CP+0.6M Urea; Lane 4: GST-CP+0.3M Urea; Lane 5: GST-CP+0.1M Urea; Lane 6: GST-CP+0.05M Urea; Lane 7: GST-CP+0.025M Urea; Lane 8: GST-CP+0.012M Urea. Lane St: Precision Plus Protein Standard (Bio-rad).
(TIF)

S3 Fig 12% SDS-PAGE of lysates from *E. coli* SHuffle. Pellet and supernatant obtained after centrifugation of equal amounts of lysate from *E*. *coli* SHuffle trasformed with wtCP and mut CP were applied as: Lane 1: supernatant of *E*. *coli* SHuffle trasformed with wtCP; Lane 2: supernatant of *E*. *coli* SHuffle trasformed with mutCP; Lane 3: pellet of *E*. *coli* SHuffle trasformed with wtCP; Lane 4: pellet of *E*. *coli* SHuffle trasformed with mutCP. Lane St: Precision Plus Protein Standard (Bio-rad).
(TIF)

S4 Fig Alignment of several cerato-platanin proteins from different fungi. Alignment was performed with ClustalW by MEGA 5.2.1. Invariable residues are marked with asterisks (*), conserved residues with colons (:). The N-terminal secretion signal sequences of all proteins were removed to optimize the alignment. Residues cited in this paper are shaded in green. The fungal protein used are from: *Trichoderma atroviride* Epl1 (G9IS53), Epl1 (A0PCX2) and Epl2 (G9MXR6); *Botrytis cinerea* (BcSpl1, 154320365); *Trichoderma virens* (SM1, Q0R411); *Phaeosphaeria nodorum* (Snodprot1, O74238); *Coccidioides immitis* (CS-AG; Q1E8D2); *Taiwanofungus camphoratus* (Aca1; Q6J935); *Moniliophthora perniciosa* MpCP1 (B2C3H7), MpCP3 (B2C3I1), MpCP2 (B2C3H9); *Neosartorya fumigate* (Aspf15, O60022); *Ceratocystis platani* (CP, P71802), *Ceratocystis populicola* (Pop1, 121624694); *Leptosphaeria maculans* (SP1, Q8J0U4).
(TIF)
