# Supplementary material for: A single amino acid mutation affects elicitor and expansins-like activities of cerato-platanin, a non-catalytic fungal protein
Source: PLoS One. 2017 May 25;12(5):e0178337. doi: 10.1371/journal.pone.0178337 (PMC5444802; doi:10.1371/journal.pone.0178337)
Supplement: S1 Table — (DOCX) [file pone.0178337.s001.docx]

| **Primers** | **Sequence** |
| --- | --- |
| **pGEX_5’** | GGGCTGGCAAGCCACGTTTGGTG |
| **pGEX_3’** | CCGGGAGCTGCATGTGTCAGAGG |
| C**P -Ala For** | CTTTATCAGGGGTGTTG**C**CAGTGGCAGAGGTGG |
| **CP-Ala Rev** | CCACCTCTGCCACTG**G**CAACACCCCTGATAAAG |

**Table S1: Primers used in molecular cloning and mutation of the *cp* gene**

pGEX_5’ and pGEX_3’ were used for cloning of *cp* gene;

CP -Ala For and CP-Ala Rev were used for mutation of *cp* gene
