# Supplementary figures and images for: A single amino acid mutation affects elicitor and expansins-like activities of cerato-platanin, a non-catalytic fungal protein
Source: PLoS One. 2017 May 25;12(5):e0178337. doi: 10.1371/journal.pone.0178337 (PMC5444802; doi:10.1371/journal.pone.0178337)

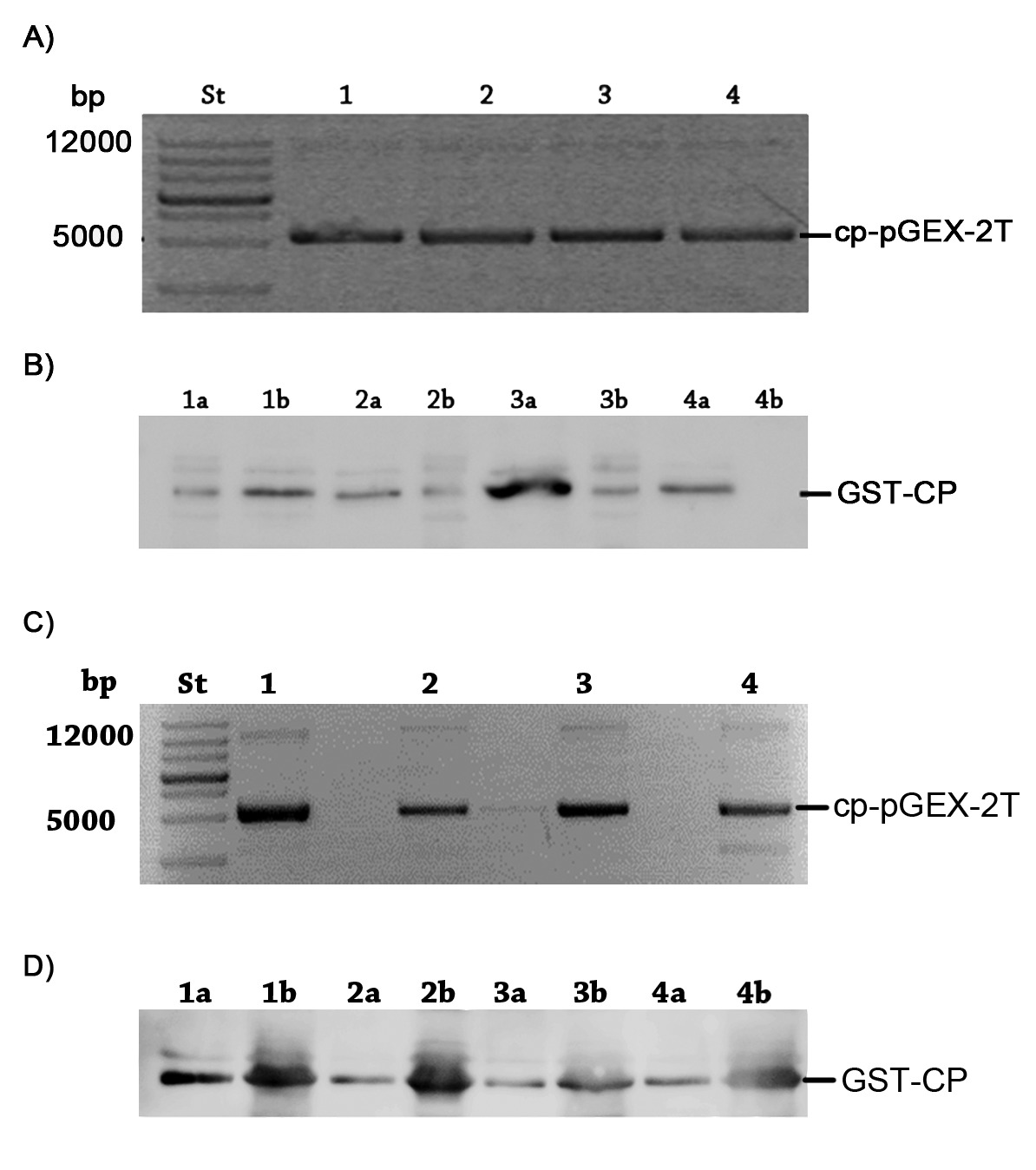

Supplement: S1 Fig — A-B) wtCP: A) 1% Agarose gel electrophoresis. Line: (St)1 Kb Plus DNA Ladder; (1–4) colony number. B) Western blot analysis probed with anti-CP of E. coli SHuffle lysate of 1–4 colonies; (a) insoluble fraction, (b) soluble fraction.C-D) mutCP: C) 1% Agarose gel electrophoresis. Line: (St)1 Kb Plus DNA Ladder; (1–4) colony number. D) Western blot analysis probed with anti-CP of E. coli SHuffle lysate of 1–4 colonies; (a) soluble fraction, (b) insoluble fraction. (TIF) [file pone.0178337.s002.tif]

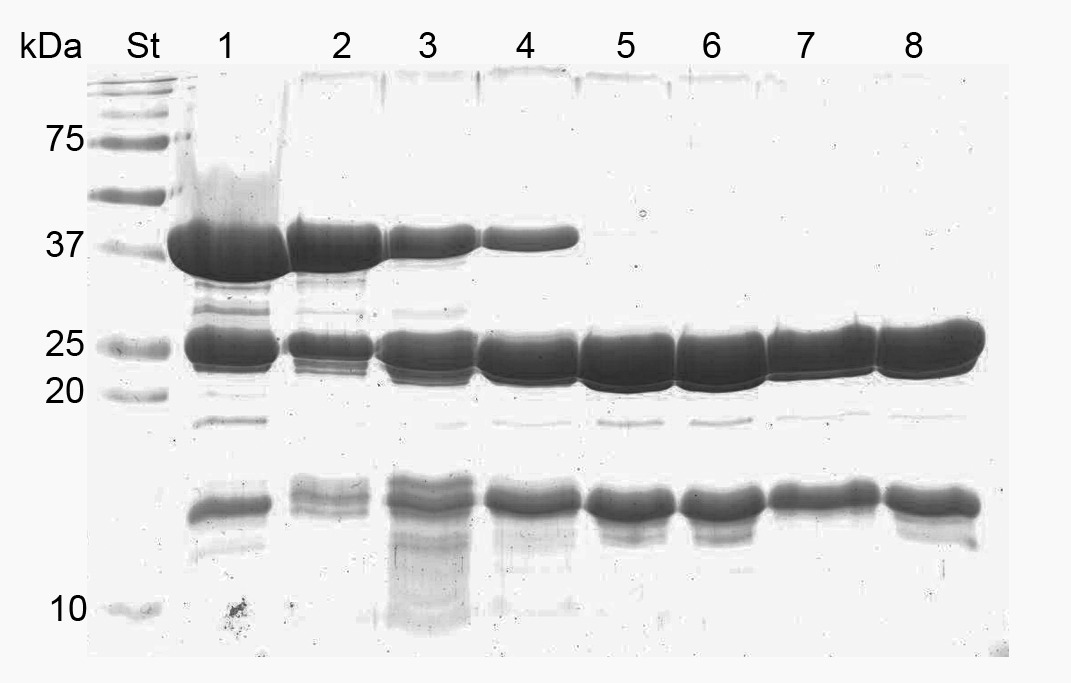

Supplement: S2 Fig — Lane 1: GST-CP+0M Urea; Lane 2: GST-CP+1M Urea; Lane 3: GST-CP+0.6M Urea; Lane 4: GST-CP+0.3M Urea; Lane 5: GST-CP+0.1M Urea; Lane 6: GST-CP+0.05M Urea; Lane 7: GST-CP+0.025M Urea; Lane 8: GST-CP+0.012M Urea. Lane St: Precision Plus Protein Standard (Bio-rad). (TIF) [file pone.0178337.s003.tif]

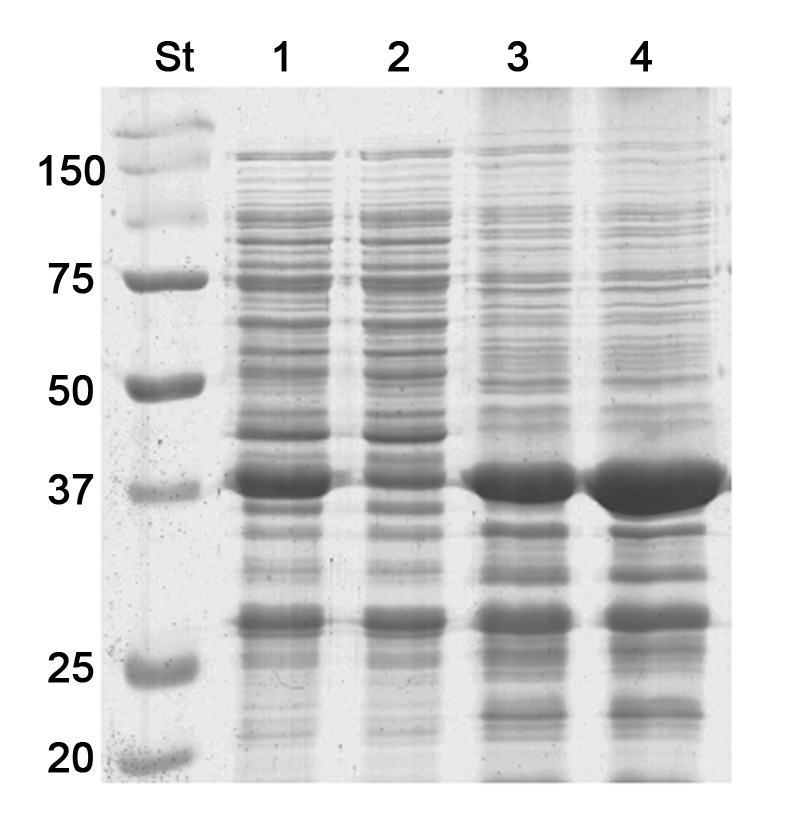

Supplement: S3 Fig — Pellet and supernatant obtained after centrifugation of equal amounts of lysate from E. coli SHuffle trasformed with wtCP and mut CP were applied as: Lane 1: supernatant of E. coli SHuffle trasformed with wtCP; Lane 2: supernatant of E. coli SHuffle trasformed with mutCP; Lane 3: pellet of E. coli SHuffle trasformed with wtCP; Lane 4: pellet of E. coli SHuffle trasformed with mutCP. Lane St: Precision Plus Protein Standard (Bio-rad). (TIF) [file pone.0178337.s004.tif]

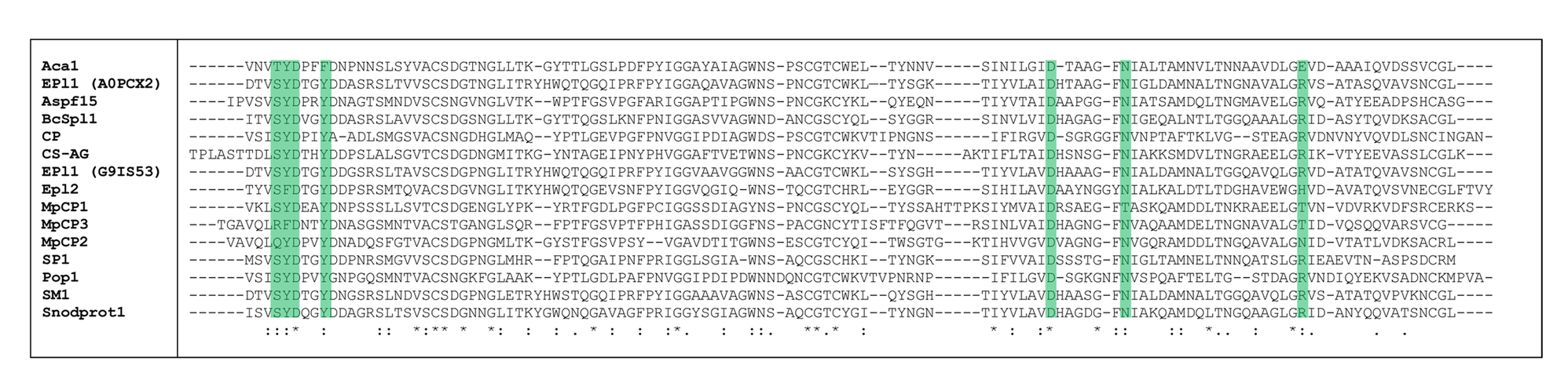

Supplement: S4 Fig — Alignment was performed with ClustalW by MEGA 5.2.1. Invariable residues are marked with asterisks (*), conserved residues with colons (:). The N-terminal secretion signal sequences of all proteins were removed to optimize the alignment. Residues cited in this paper are shaded in green. The fungal protein used are from: Trichoderma atroviride Epl1 (G9IS53), Epl1 (A0PCX2) and Epl2 (G9MXR6); Botrytis cinerea (BcSpl1, 154320365); Trichoderma virens (SM1, Q0R411); Phaeosphaeria nodorum (Snodprot1, O74238); Coccidioides immitis (CS-AG; Q1E8D2); Taiwanofungus camphoratus (Aca1; Q6J935); Moniliophthora perniciosa MpCP1 (B2C3H7), MpCP3 (B2C3I1), MpCP2 (B2C3H9); Neosartorya fumigate (Aspf15, O60022); Ceratocystis platani (CP, P71802), Ceratocystis populicola (Pop1, 121624694); Leptosphaeria maculans (SP1, Q8J0U4). (TIF) [file pone.0178337.s005.tif]
